# Tracheal Intubation Without the Use of Neuromuscular Blocking Agents

**DOI:** 10.5812/aapm.3478

**Published:** 2012-07-10

**Authors:** Smita Prakash

**Affiliations:** 1 Department of Anaesthesia and Intensive Care, Vardhman Mahavir Medical College and Safdarjang Hospital, New Delhi, India

**Keywords:** Alfentanil, Remifentanil, Propofol, Intubation

*Dear Editor,*

I read with keen interest the article by Imani *et al.* (1) in which the authors compare tracheal intubating conditions following intravenous (IV) administration of remifentanil 5 μg/kg or alfentanil 50 μg/kg followed by induction of anesthesia with propofol 2 mg/kg in 100 adult patients premedicated with IV midazolam 1 mg. Intubating conditions were assessed (scoring 1 to 4) by ease of laryngoscopy, patency of vocal cords, jaw relaxation, and limb movement. Airway reaction to tracheal intubation is an important consideration while assessing intubating conditions. Viby-Mogensen *et al*. (2) describe a qualitative scoring system graded as excellent, good or poor, respectively, comprising of five variables:

1. Laryngoscopy (easy, fair, difficult)
2. Vocal cord position (abducted, intermediate, closed)
3. Vocal cord movement (none, moving, closing)
4. Coughing (none, diaphragmatic, sustained, > 20 s)
5. Movement of the limbs (none, slight, vigorous)

Intubating conditions are regarded as excellent (all qualities are excellent), good (all qualities are either excellent or good), and poor (the presence of a single quality listed under poor) (2). They further regard excellent or good intubating conditions as clinically acceptable; and poor intubating conditions as clinically not acceptable. It would be interesting to compare the two narcotic regimens (remifentanil *vs.* alfentanil) for tracheal intubation without neuromuscular blocking agents (NMBAs) with regard to the incidence of coughing.

Tracheal intubation is associated with hemodynamic changes. In this regard, the use of remifentanil 5 μg/kg would be expected to cause significant hypotension and/or bradycardia. It would be useful to know the incidence of hypotension or bradycardia and also if there was need for administration of vasopressors or atropine.

I am intrigued by two statements in the article that are in contradistinction:

1. The combination of remifentanil with propofol may also be advantageous in cases of long and difficult intubation, wherein it may not only be possible to inspect the airway with the laryngoscope.
2. Thus, this method is best avoided in patients with high Mallampati grades or airway difficulties.

The technique of tracheal intubation without the use of NMBAs enables us to assess the airway by laryngoscopy. This technique may be useful in cases of both predicted and unexpected difficult intubation, in cases where NMBAs are either contraindicated (e.g. myopathies) or not required to facilitate surgical access (3).

## References

[1] Imani F, Alebouyeh MR, Taghipour-Anvari Z, Faiz SHR (2011). Use of Remifentanil and Alfentanil in Endotracheal Intubation: A Comparative Study. Anesth Pain.

[2] Viby-Mogensen J, Engbaek J, Eriksson LI, Gramstad L, Jensen E, Jensen FS (1996). Good clinical research practice (GCRP) in pharmacodynamic studies of neuromuscular blocking agents. Acta Anaesthesiol Scand.

[3] Prakash S, Arora D, Bhartiya V, Singh R (2006). A combination of fentanyl-midazolam-propofol provides better intubating conditions than fentanyl-lignocaine-propofol in the absence of neuromuscular blocking agents. Acta Anaesthesiol Scand.

